# Association between Obesity and Parental Weight Status in Children and Adolescents

**DOI:** 10.4274/jcrpe.3790

**Published:** 2017-06-01

**Authors:** Maryam Bahreynian, Mostafa Qorbani, Bita Moradi Khaniabadi, Mohammad Esmaeil Motlagh, Omid Safari, Hamid Asayesh, Roya Kelishadi

**Affiliations:** 1 Research Institute for Primordial Prevention of Non Communicable Disease, Child Growth and Development Research Center, Department of Pediatrics, Isfahan University of Medical Sciences, Isfahan, Iran; 2 Non-Communicable Diseases Research Center, Alborz University of Medical Sciences, Karaj, Iran; 3 Chronic Diseases Research Center, Endocrinology and Metabolism Population Sciences Institute, Tehran University of Medical Sciences, Tehran, Iran; 4 Department of Pediatrics, Ahvaz Jundishapur University of Medical Sciences, Ahvaz, Iran; 5 Department of Medical Emergencies, Qom University of Medical Sciences, Qom, Iran

**Keywords:** overweight, obesity, body mass index, children and adolescents, parents

## Abstract

**Objective:**

This study aims to assess the relationship between body mass index (BMI) of children and that of their parents in a nationally-representative sample of Iranian population.

**Methods:**

This cross-sectional nationwide study was conducted in 2011-2012 among 6-18-year-old students and their parents living in 30 provinces of Iran. Socio-demographic information was collected. The BMI values of the children/adolescents were categorized according to the World Health Organization reference curves. Association between parental and student weight status was examined using ordinal regression models after adjustment for potential confounders.

**Results:**

Overall, 23043 children and adolescents and one of their parents participated in this study (50.7% boys, 73.4% urban status). Mean age of the subjects was 12.55±3.31 years. Mean BMI values of parents and children/adolescents were 27.0±4.57 and 18.8±4.4 kg/m2, respectively. After adjusting for confounders, overweight and/or obesity in students of both genders was found to be significantly associated with parental overweight and/or obesity. In those students who had obese parents, the odds ratio (OR) of being obese was 2.79 for boys [OR=2.79; 95% confidence interval (CI)=2.44-3.20] and 3.46 for girls (OR=3.46; 95% CI=3.03-3.94) compared to their peers with normal-weight parents. Boys with overweight parents were 1.7 times more overweight than their counterparts with normal-weight parents (OR=1.70; 95% CI=1.15-1.92). Similarly, girls who had overweight parents were more overweight compared to those with normal-weight parents (OR=2.00; 95% CI=1.77-2.25).

**Conclusion:**

Our findings highlight the importance of the shared family environment as a multi-factorial contributor to the childhood obesity epidemic and the necessity of implementing family-centered preventive programs.

## What is already known on this topic?

Parental weight is one of the predictors of obesity in children and adolescents. There is an association between parental body mass index and child birth weight.

## What this study adds?

Children from families with obese parents were at a significantly higher risk of obesity compared to children with normal-weight parents. Overweight and/or obesity in children of both genders was significantly associated with parental overweight and/or obesity.

## INTRODUCTION

Overweight and obesity in childhood usually track to adulthood. They are associated with several complications and increase the risk of morbidity and mortality later in life (1).

The prevalence of overweight and obesity is increasing in both developed and developing countries (2). The overweight/obesity prevalence has doubled and tripled in pre-school and primary school-aged children, respectively (3). Among developing societies, Eastern Europe and the Middle East have the highest prevalence of childhood overweight (4).

In the Middle East, a high frequency of overweight and/or obesity was documented in adolescents living in Kuwait (5) and Qatar (6). The prevalence of overweight and/or obesity in children and adolescents is on the increase in many developing countries, such as Iran (7). In our previous nationwide study, the prevalence rate of general and abdominal obesity in 6-18 years Iranian students was 11.89% (13.58% of boys vs. 10.15% of girls) and 19.12% (20.41% of boys vs. 17.79% of girls), respectively (8).

Excess weight/obesity is a multi-factorial disorder and derives from two different origins, namely, genetic and environmental factors. However, the relative contributing role of genetic susceptibility and environmental factors to development of obesity is not clear (9). A great number of previous studies have indicated that childhood and adolescent overweight and obesity are linked to obvious familial aggregation, as a result of complex interaction between genetic and environmental effects (10,11).

During recent decades, a number of studies have shown the association between parental body mass index (BMI) and child birth weight (12). In addition, studies indicate the higher impact of parental BMI on the severity of weight gain from childhood to adolescence (13).

Parental weight has been shown as an important predictor for obesity development in children and adolescents. Few studies are available regarding the association between parental and child obesity (14). The present study aims to examine the relationship of parental BMI with overweight and obesity in children/adolescents in a nationally representative sample of Iranian population.

## METHODS

The Childhood and Adolescence Surveillance and Prevention of Adult Noncommunicable Disease-IV Study was performed in rural and urban regions of 30 provinces of Iran in 2011-2012. The methodology of the study has been published in detail (15). In brief, students were selected from elementary, middle, and high schools by multistage, cluster random sampling method. Stratification was done according to the level of the schools (elementary, middle, and high school) and place of residence (urban, rural). The total child/adolescent sample size was calculated as 25000 students (48 clusters of 10 students in each province) and 23043 students participated in the survey.

Trained health care professionals conducted the physical examination under standard protocols by using calibrated instruments. These professionals were selected from health staff working in the health system in each province (one person in each province, a total of 30 professionals) and attended a 3-day educational workshop on measurement of anthropometric indices according to standard protocols.

Weight was measured with the subject in light clothing, to the nearest 0.1 kg. Standing height was recorded without shoes to the nearest 0.1 cm. BMI was calculated as weight (Kg) divided by height in square meters (m^2^). BMI categories were defined according to the World Health Organization (WHO) reference curves for different age and gender groups (16). The subjects were classified as underweight (BMI <5^th^ percentile), normal weight (BMI between 5^th^-85^th^ percentiles), overweight (BMI between 85^th^-95^th^ percentiles), and obese (BMI ≥85^th^-95^th^ percentiles). Parents were asked to report their weight and height. Parental BMI was calculated as underweight (BMI <18.5), normal-weight (18.5< BMI <24.9), overweight (BMI ≥25-29.9), and obese (BMI ≥30). Socio-demographic information including parental education and occupation, age of the subject, living area (urban vs. rural), and number of people living in the house were also collected.

### Statistical Analysis

All analyses were conducted using survey analysis method in STATA software. Categorical and continuous data were presented as numbers (percentages) and means standard deviation (SD), respectively. The weight status of the children/adolescents was analyzed as an ordinal outcome variable. Parental weight status was categorized into four groups (underweight, normal weight, overweight, and obese), which was investigated as an ordinal response variable. Univariate analysis was used to examine the relationship of each independent variable to outcome of interest. Goodman and Kruskal’s gamma and Pearson’s chi-square statistic were used to determine the association between the weight status and participants’ characteristics. Pearson’s correlation test was used to investigate the relationship between continuous variables and BMI values. A variable univariately associated with the outcome was added to the preliminary multivariate model for the outcome.

Ordinal regression models were applied to estimate the odds ratios (ORs) and 95% confidence intervals (CIs) of obesity by parental weight status, adjusted for potential confounders. These models were tested using a full likelihood ratio test comparing the fitted location model to a model varying location parameters. Hence, separate binomial logistic regressions were run on cumulative dependent variables. Boys and girls were analyzed separately due to gender differences in weight status. Two sets of potential confounders were used in the adjusted models. Model 1 was adjusted for some characteristics of children/adolescents including birth weight, gender, living area, and age. Model 2 was further adjusted for potential predictors which were related to their family such as parental education and occupation, type of house and school, and number of the household. All the tests were two-sided and the significance level was set as 0.05.

## RESULTS

In total, 23043 students (50.8% boys) completed the study. The mean and SD for age of the subjects was 12.55 (3.31) years. Overall, 73.4% of the study participants were from urban areas and 79.4% of families lived in their private house. Parental mean weight (SD) was 70.9±13.8 kg and that of the students was 42.50±17.0 kg. 17% of fathers and 9.4% of mothers had university degrees. Ninety percent (90.9%) of children/adolescents were students of public schools. About half of fathers (44.38%) were laborers or white collar workers and 88.3% of the mothers were housewives. Nearly half of families in the study owned personal cars and personal computers. The basic and demographic characteristics of the study participants are given in Table 1. Since the weight status was statistically significantly different between boys and girls, simple bivariate analyses were performed for boys (n=11752) and girls (n=11364) separately.

The prevalence of overweight/obesity was 15.1% and 65.6% among the students and the parents, respectively. Mean (SD) BMI was18.81±4.43 and 27.0±4.57 kg/m^2^ for the children/adolescents and the parents, respectively. Overall, 5.0% of children/adolescents were categorized as obese, 10.1% were overweight, and 5.0% were underweight. There was an association between children/adolescents’ weight and parental weight status (p<0.001 for both genders). We found that 0.8% of boys with underweight parents were overweight or obese. 88.8% of children/adolescents with overweight parents and 67.0% of children/adolescents with obese parents were overweight or obese.

Table 2 indicates the multivariate regression models for the association and obesity in children/adolescents and parental weight, adjusted for potential confounders. The analyses were stratified by gender. The prevalence of obesity was high among parents. In models 1-2, childhood and/or adolescent obesity was significantly associated with parental obesity in both genders, after adjusting for confounders. Parental overweight increased the odds of obesity among children and adolescents and strong associations were found between overweight and obese parents and the weight of their offspring (p<0.05). Boys were 2.79 times more likely to be obese if their parents were obese compared to boys with normal weight parents (OR=2.79; 95% CI=2.44-3.20). Similarly, girls who had obese parents were more likely to be obese than their peers who had normal-weight parents (OR=3.46, 95% CI=3.03-3.94). Boys with overweight parents were 1.7 times more overweight than their counterparts with normal-weight parents (OR=1.70; 95% CI=1.15-1.92). In the same way, girls who had overweight parents were more overweight compared to those with normal weight parents (OR=2.00; 95% CI=1.77-2.25).

## DISCUSSION

The current study is one of the first of its kind to explore the association of the weight status of a large population-based sample of Iranian children with that of their parents at national level. We found that children from families with obese parents were at a significantly higher risk of obesity compared to children of normal-weight parents.

Both genetic and environmental factors contribute to childhood obesity (17,18). Some environmental factors including parental overweight, shared family lifestyle, dietary habits, and socio-economic status (SES) are linked to childhood overweight (7,18,19,20,21). Previous studies have indicated that low SES families have little access to healthy foods; therefore, their consumption of high-calorie, low nutrient foods is higher than that of high SES groups (22,23). Parental education has also been reported to be inversely related to child excess weight and studies have shown a higher prevalence of overweight and/or obesity in children of parents with a low educational level (24,25,26). Previous studies across 11 European countries have indicated that low maternal education could yield a substantial risk of early childhood obesity (27). In another study, it was reported that children of better educated mothers had a more favorable growth pattern, namely, lower overweight and obesity rates (in the UK and Sweden), and lower stunting and underweight rates (in rural China) (28). It seems that maternal education has a more substantial effect on child weight status, because mothers spent a longer time with their children than the fathers and are usually the person who prepares the food (27). Also, there is a more direct interaction between the children and their mothers than their fathers (29). The inverse association between parental education and child obesity could possibly affect life-style, dietary intake, as well as the SES position (24,25,30).

One of the most important components of family context is parental weight status, which has been reported to be an important predictor of overweight and obesity in children and adolescents (14,17,18,30). However, to date, the potential association with parental weight has not been extensively investigated.

In the current study, obese parents were more likely to have overweight or obese children, compared to normal-weight parents. These results are consistent with previous studies (14,18,30) and suggest that parental excess weight has an important role on child BMI.

Overweight parents are considered as risk factors for overweight/obesity of their offspring (1,31). The association between overweight children and parental excess weight represents both gene and environment interactions (19). Thus, the increasing risk of childhood or adolescent overweight in individuals with obese parents might be due to their genetics or their living in the same environment. Furthermore, children usually imitate their parents. Therefore, eating habits and family lifestyle could have an influence on child eating behavior. Unfavorable parental eating patterns (including higher consumption of fried, fast foods, sweets) and a sedentary lifestyle such as low physical activity and prolonged TV and computer time might increase the risk of overweight and obesity in both parents and their children (18).

Our study is the first national study conducted on a very large sample to determine the association between an overweight status in children and parental weight status. The study has some limitations which need to be addressed. As a cross-sectional study, we cannot infer cause and effect relationships, thus the association of parental BMI and child weight status needs to be confirmed in prospective studies. The other limitation is the self-reported heights and weights of parents, which may have decreased the accuracy of these data. Estimated obesity prevalence in parents might be affected by underreporting of weight due to the social desirability. However, a number of studies in our community have shown that self-reported anthropometric measurements are reliable (32). Our findings also highlight the importance of asking about family history of overweight and/or obesity by family physicians and primary care practitioners to be able to assess the risk of overweight in children.

In conclusion, our findings highlight the importance of the shared family environment as a multi-factorial contributor to the childhood obesity epidemic as well as the necessity of implementing family-centered preventive programs.

## Figures and Tables

**Table 1:**
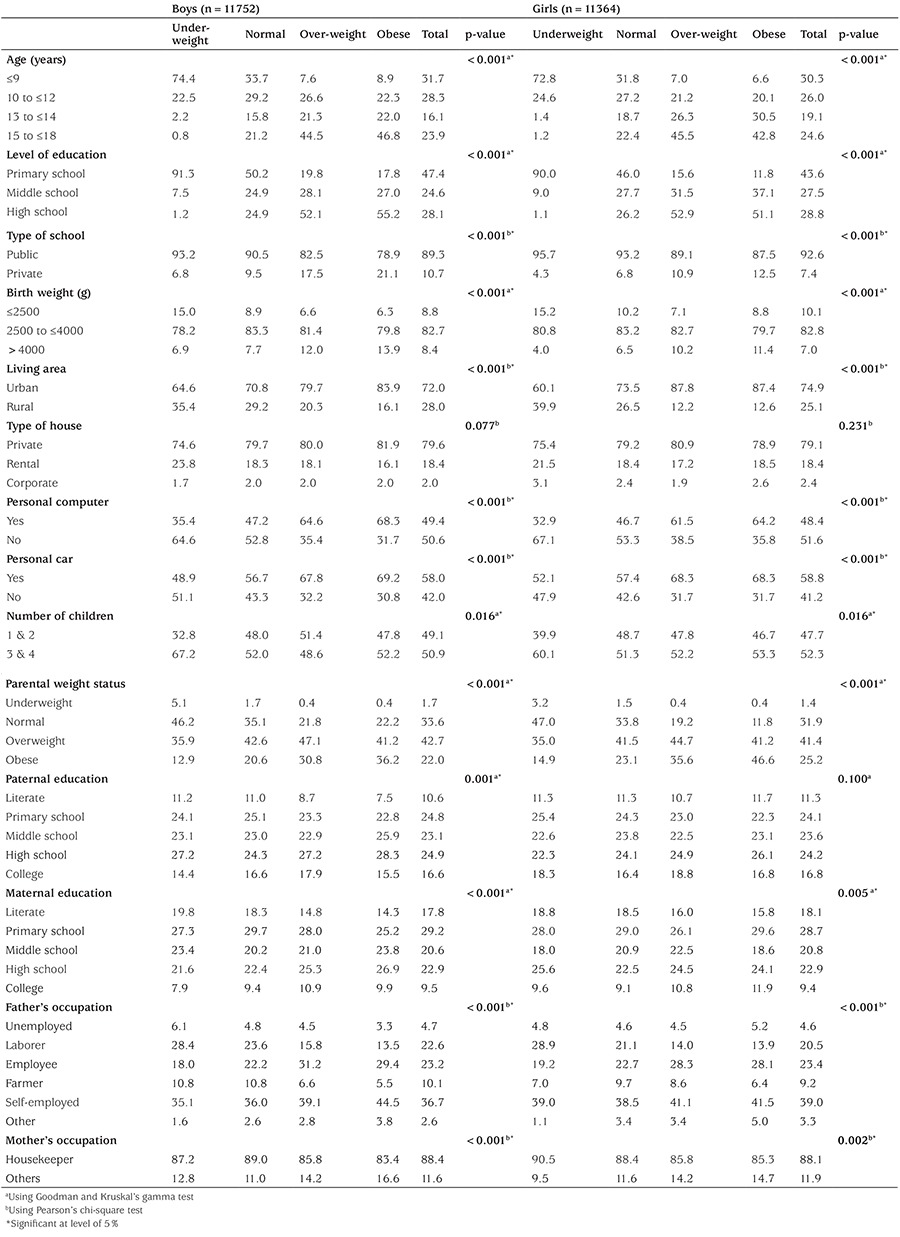
Descriptive characteristics of participants: The weight disorder survey of the CASPIAN-IV Study (Column %)

**Table 2:**
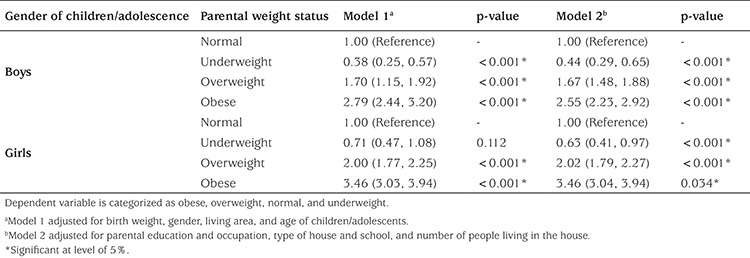
Adjusted odds ratios (95% confidence intervals) for the association of children/adolescent obesity with parental weight status by gender: The weight disorder survey of the CASPIAN-IV Study

## References

[1] Ebbeling CB, Pawlak DB, Ludwig DS (2002). Childhood obesity: public-health crisis, common sense cure. Lancet.

[2] Rennie KL, Jebb SA (2005). Prevalence of obesity in Great Britain. Obes Rev.

[3] Ogden CL, Flegal KM, Carroll MD, Johnson CL (2002). Prevalence and trends in overweight among US children and adolescents, 1999-2000. JAMA.

[4] Kelishadi R (2007). Childhood overweight, obesity, and the metabolic syndrome in developing countries. Epidemiol Rev.

[5] Al-Isa AN (2004). Body mass index, overweight and obesity among Kuwaiti intermediate school adolescents aged 10-14 years. Eur J Clin Nutr.

[6] Bener A (2006). Prevalence of obesity, overweight, and underweight in Qatari adolescents. Food Nutr Bull.

[7] Bahreynian M, Kelishadi R, Qorbani M, Motlagh ME, Kasaeian A, Ardalan G, Rad TA, Najafi F, Asayesh H, Heshmat R (2015). Weight disorders and anthropometric indices according to socioeconomic status of living place in Iranian children and adolescents: The CASPIAN-IV study. J Res Med Sci.

[8] Esmaili H, Bahreynian M, Qorbani M, Motlagh ME, Ardalan G, Heshmat R, Kelishadi R (2015). Prevalence of General and Abdominal Obesity in a Nationally Representative Sample of Iranian Children and Adolescents: The CASPIAN-IV Study. Iran J Pediatr.

[9] Barness LA, Opitz JM, Gilbert‐Barness E (2007). Obesity: genetic, molecular, and environmental aspects. Am J Med Genet A.

[10] Xi B, Mi J, Duan JL, Yan SJ, Cheng H, Hou DQ, Zhao XY (2009). [Familial clustering of obesity and the role of lifestyle factors among children in Beijing]. Zhonghua Yu Fang Yi Xue Za Zhi.

[11] Lobstein T, Baur L, Uauy R, IASO International Obesity TaskForce (2004). Obesity in children and young people: a crisis in public health. Obes Rev.

[12] Sandovici I, Smith NH, Nitert MD, Ackers-Johnson M, Uribe-Lewis S, Ito Y, Jones RH, Marquez VE, Cairns W, Tadayyon M, O’Neill LP, Murrell A, Ling C, Constância M, Ozanne SE (2011). Maternal diet and aging alter the epigenetic control of a promoter-enhancer interaction at the Hnf4a gene in rat pancreatic islets. Proc Natl Acad Sci U S A..

[13] Trost SG, Kerr L, Ward DS, Pate RR (2001). Physical activity and determinants of physical activity in obese and non-obese children. Int J Obes Relat Metab Disord.

[14] Shafaghi K, Shariff ZM, Taib MN, Rahman HA, Mobarhan MG, Jabbari H (2014). Parental body mass index is associated with adolescent overweight and obesity in Mashhad, Iran. Asia Pac J Clin Nutr.

[15] Kelishadi R, Motlagh ME, Bahreynian M, Gharavi MJ, Kabir K, Ardalan G, Safari O, Qorbani M (2015). Methodology and Early Findings of the Assessment of Determinants of Weight Disorders among Iranian Children and Adolescents: The Childhood and Adolescence Surveillance and PreventIon of Adult Noncommunicable Disease-IV Study. Int J Prev Med.

[16] WHO Multicentre Growth Reference Study Group (2006). WHO Child Growth Standards based on length/height, weight and age. Acta Paediatr Suppl.

[17] Stettler N, Tershakovec AM, Zemel BS, Leonard MB, Boston RC, Katz SH, Stallings VA (2000). Early risk factors for increased adiposity: a cohort study of African American subjects followed from birth to young adulthood. Am J Clin Nutr.

[18] Jiang MH, Yang Y, Guo XF, Sun YX (2013). Association between child and adolescent obesity and parental weight status: a cross-sectional study from rural North China. J Int Med Res.

[19] Davison KK, Birch LL (2002). Obesigenic families: parents’ physical activity and dietary intake patterns predict girls’ risk of overweight. Int J Obes Relat Metab Disord.

[20] Semmler C, Ashcroft J, Jaarsveld CH, Carnell S, Wardle J (2009). Development of overweight in children in relation to parental weight and socioeconomic status. Obesity (Silver Spring).

[21] Bahreynian M, Motlagh ME, Qorbani M, Heshmat R, Ardalan G, Kelishadi R (2015). Prevalence of Growth Disorders in a Nationally Representative Sample of Iranian Adolescents According to Socioeconomic Status: The CASPIAN-III Study. Pediatr Neonatol.

[22] Irala-Estevez JD, Groth M, Johansson L, Oltersdorf U, Prattala R, Martinez-Gonzalez MA (2000). A systematic review of socio-economic differences in food habits in Europe: consumption of fruit and vegetables. Eur J Clin Nutr.

[23] Wardle J, Guthrie C, Sanderson S, Birch L, Plomin R (2001). Food and activity preferences in children of lean and obese parents. Int J Obes Relat Metab Disord.

[24] Cribb VL, Jones LR, Rogers IS, Ness AR, Emmett PM (2011). Is maternal education level associated with diet in 10-year-old children?. Public Health Nutr.

[25] Vereecken CA, Keukelier E, Maes L (2004). Influence of mother’s educational level on food parenting practices and food habits of young children. Appetite.

[26] Langnase K, Mast M, Muller MJ (2002). Social class differences in overweight of prepubertal children in northwest Germany. Int J Obes Relat Metab Disord.

[27] Ruiz M, Goldblatt P, Morrison J, Porta D, Forastiere F, Hryhorczuk D, Antipkin Y, Saurel-Cubizolles MJ, Lioret S, Vrijheid M, Torrent M, Iñiguez C, Larrañaga I, Bakoula C, Veltsista A, Eijsden M, Vrijkotte TG, Andrýsková L, Dušek L, Barros H, Correia S, Järvelin MR, Taanila A, Ludvigsson J, Faresjö T, Marmot M, Pikhart H (2016). Impact of Low Maternal Education on Early Childhood Overweight and Obesity in Europe. Paediatr Perinat Epidemiol.

[28] Lakshman R, Zhang J, Zhang J, Koch FS, Marcus C, Ludvigsson J, Ong KK, Sobko T (2013). Higher maternal education is associated with favourable growth of young children in different countries. J Epidemiol Community Health.

[29] Craig LC, McNeill G, Macdiarmid JI, Masson LF, Holmes BA (2010). Dietary patterns of school-age children in Scotland: association with socio-economic indicators, physical activity and obesity. Br J Nutr.

[30] Lazzeri G, Pammolli A, Pilato V, Giacchi MV (2011). Relationship between 8/9-yr-old school children BMI, parents’ BMI and educational level: a cross sectional survey. Nutr J.

[31] Würbach A, Zellner K, Kromeyer-Hauschild K (2009). Meal patterns among children and adolescents and their associations with weight status and parental characteristics. Public Health Nutr.

[32] Engstrom JL, Paterson SA, Doherty A, Trabulsi M, Speer KL (2003). Accuracy of self-reported height and weight in women: an integrative review of the literature. J Midwifery Womens Health.

